# Sec8 specifically interacts with the PDZ2 domain of synapse associated protein 102 (SAP102)

**DOI:** 10.3389/fcell.2023.1254611

**Published:** 2023-10-02

**Authors:** Katharina Korbula, Iana Hammerschmid, Johannes Lesigang, Gang Dong

**Affiliations:** ^1^ Max Perutz Labs, Vienna Biocenter Campus (VBC), Vienna, Austria; ^2^ Medical University of Vienna, Center for Medical Biochemistry, Vienna, Austria

**Keywords:** crystal structure, exocyst, protein interaction, SAP102, Sec8, vesicle trafficking

## Abstract

The exocyst is an evolutionarily conserved protein complex tethering secretory vesicles before their docking and fusion with the plasma membrane. The complex also plays important roles in cell migration, synaptogenesis, and neurite outgrowth. One of its subunits, Sec8, was reported to interact with two major synaptic scaffolding proteins SAP102 and PSD-95 that share high sequence homology and contain three PDZ domains at their N-terminal region. The interaction is via the binding of the C-terminal ITTV motif in Sec8 to the PDZ domains of the two synaptic proteins. However, it remains elusive to which PDZ domain(s) Sec8 binds and how their interaction occurs. Here we reported a 2.5 Å resolution crystal structure of the C-terminal half of rat Sec8 containing the ITTV motif. The structure shows that Sec8 contains an enormously long helix at its C-terminus, which bears a unique long “spacer” of 14 residues to bridge the ITTV motif to the compact core of Sec8. We found that Sec8 preferentially binds PDZ2 over PDZ1 and PDZ3 of SAP102. Deletion of the spacer completely abolished the binding of Sec8 to SAP102. Overall, our structural studies, biochemical data and modeling analyses altogether provide an explanation for how Sec8 interacts with SAP102.

## Introduction

Synapse-associated protein 102 (SAP102) and postsynaptic density 95 (PSD-95) are two major cytoskeleton proteins in the postsynaptic density (PSD). They are both membrane-associated guanylate kinases (MAGUKs) and share 56% identity and 68% similarity in their primary sequences. The two proteins have a common domain organization, consisting of three PDZ domains in their N-terminal half, a central Src homology 3 (SH3) domain, and a C-terminal guanylate kinase (GuKc) domain. Previous studies showed that the localization of PSD-95 and SAP102 to neuronal synapses is mediated by the exocyst complex; they together regulate the sorting and trafficking of *N*-methyl-D-aspartate receptors (NMDARs) from the endoplasmic reticulum (ER) and the Golgi apparatus to the synapse (Riefler et al., 2003; Sans et al., 2003).

The exocyst is an octameric protein complex tethering secretory vesicles to the plasma membrane prior to membrane fusion (TerBush and Novick, 1995; TerBush et al., 1996). The interaction of SAP102 and PSD-95 with the exocyst is via one of its subunits, Sec8, which contains a consensus ITTV motif at the C-terminus for PDZ binding. Crystal structures of individual PDZ domains of SAP102 and PSD-95 in both apo- and ligand-bound forms have been reported (Doyle et al., 1996; Elkins et al., 2007; Camara-Artigas et al., 2010; Sainlos et al., 2011; Bach et al., 2012; Sainlos et al., 2013; Zeng et al., 2016; Camara-Artigas et al., 2019; Rodzli et al., 2020; Fukata et al., 2021). Intriguingly, however, despite the high similarity in the structures of these PDZ domains, Sec8 binds the three PDZ domains with different affinities (Riefler et al., 2003; Sans et al., 2003). To find out how Sec8 interacts with SAP102, particularly to which PDZ domain(s) Sec8 binds, we carried out extensive structural, biophysical and biochemical analyses on the two proteins and their interactions.

We have determined a 2.5 Å resolution crystal structure of the C-terminal half of rat Sec8. In the structure, we found that the ITTV motif is attached to an enormously long α-helix. The C-terminal half of this helix, which we named “spacer,” protrudes from the helically bundled core of the Sec8 structure. The spacer is sandwiched between the PDZ-binding site and the rest of Sec8. Using a series of truncations of SAP102, we revealed that Sec8 binds specifically to SAP102-PDZ2, but not to either PDZ1 or PDZ3. We further found that the spacer is critical for Sec8 to bind to SAP102, as deletion of it completely abolishes their interaction. Notably, this spacer is uniquely present in Sec8 but absent in all other exocyst subunits. Structural modeling analyses demonstrate that the spacer allows the ITTV motif at the C-terminal end of Sec8 to interact with SAP102-PDZ2 without causing clashes between the two proteins.

## Materials and methods

### Molecular cloning

To characterize the interaction between Sec8 and SAP102 in detail, we generated multiple truncations of SAP102 containing one or more PDZ domains. These include PDZ1 (aa144–236), PDZ2 (aa241–331), PDZ3 (aa399–493), and PDZ1-PDZ2 (aa144–331). We also cloned the C-terminal half of Sec8 (Sec8c, aa544–975) as well as an internal deletion construct of it (Sec8c-Δ958–971, aa544–975/Δ958–971) for structural studies and/or binding assays. All target DNA sequences were amplified using PCR reactions and ligated into either of the two custom vectors that provide a His_6_-SUMO tag (for SAP102) or an MBP-His_10_ tag (for Sec8) to the N-terminus of the target proteins. The two fusion tags can be cleaved off by the SENP2 and the tobacco etch virus (TEV) proteases, respectively.

### Protein expression and purification

All recombinant proteins were expressed in *Escherichia coli* (strain BL21-DE3). The cells transformed with the expression constructs were grown in Luria-Bertani (LB) medium at 37°C to an OD_600_ of 0.6–0.8, and then subjected to cold shock on ice for 30 min. Protein expression was induced by addition of 0.5 mM isopropyl β-D-1-thiogalactopyranoside (IPTG), and cell cultures were further incubated at 18°C overnight. The cells were harvested by centrifugation in a Sorvall GS3 rotor (6,000 × g, 12 min, 4°C), and the pellets were resuspended in 10 mL of lysis buffer [20 mM HEPES (pH 7.5), 100 mM NaCl, 20 mM imidazole, 10 mM β-mercaptoethanol] per L of cell culture.

Resuspended cells were lysed using an EmulsiFlex-C3 homogenizer (Avestin) at 40 bars and cell debris was pelleted by centrifugation (40,000 × g, 30 min, 4°C). The supernatant containing soluble proteins was filtered (0.45-µm pore size, Amicon) and loaded onto a Ni-HiTrap^®^ HP column pre-equilibrated with the same lysis buffer in an ÄKTA™ Pure chromatography system (Cytiva). The column with bound proteins was washed with 5× column volume (cv) of lysis buffer, and bound protein was subsequently eluted using a linear gradient concentration of imidazole (20–500 mM, 20× cv) in the same lysis buffer.

Purified His_6_-SUMO-SAP102 proteins (20–40 mL) were incubated with ∼1% (w/w) of the SENP2 protease in a sealed dialysis bag against 2–4 L of dialysis buffer containing 20 mM HEPES (pH 7.5), 100 mM NaCl, and 10 mM β-mercaptoethanol (4°C, overnight). The dialyzed samples were loaded onto a Ni-HiTrap^®^ HP column to get rid of the cleaved tag and any uncut proteins. Target proteins without tag appeared in the flow-through, which were further purified on a HiLoad^®^ 16/600 Superdex^®^ 200 pg column (GE Healthcare) with a running buffer containing 20 mM HEPES (pH 7.5), 100 mM NaCl, and 1 mM dithiothreitol (DTT). The eluted proteins were pooled and concentrated, and final concentration was determined using Ultraviolet (UV) absorbance at 280 nm. The samples were either used freshly or stored at −80°C for subsequent experiments.

Purified recombinant proteins of MBP-His_10_-Sec8c and the corresponding internally deleted construct were used directly for *in vitro* pull-down assays. For structural studies, some of the purified MBP-His_10_-Sec8c was further incubated with ∼1% (w/w) of the TEV protease in a dialysis bag against 2–4 L of dialysis buffer containing 20 mM HEPES (pH 7.5), 100 mM NaCl, and 10 mM β-mercaptoethanol (4°C, overnight). The rest of the purification procedure is the same as the aforementioned purification protocol for SAP102.

Selenomethionine (SeMet)-substituted Sec8c for *de novo* phase determination in our structural studies was expressed using M9 minimal medium supplemented with all amino acids (2 mg/mL) except for methionine. Prior to induction, L-SeMet was added to 80 mg/L, and additional threonine, lysine, phenylalanine, leucine, isoleucine, and valine were added to inhibit the methionine biosynthetic pathway (Doublie, 1997). The SeMet-substituted Sec8c protein was purified as described above for the unlabeled Sec8c protein, except for adding 15 mM β-mercaptoethanol for Ni-HiTrap^®^ HP purification and an extra purification step on a HiLoad^®^ 16/600 Superdex^®^ 200 pg column (GE Healthcare) using the running buffer containing 20 mM HEPES (pH 7.5), 100 mM NaCl, 5% (v/v) glycerol, and 10 mM DTT.

### Crystallization and structural determination

Purified Sec8c was subjected to extensive crystallization trials using several commercial crystallization kits (Hampton Research). Conditions of the initial hits were further optimized. Single rod-shaped crystals appeared with 24 h and grew to a maximum size of approximately 50 µm × 50 µm × 300 µm within a week at room temperature. The crystallization condition generating the SeMet-Sec8c crystals used for final data collection contained 0.1 M 2-(N-morpholino) ethanesulfonic acid (MES, pH 6.5), 1.75 M NaCl, 12.5% (w/v) PEG6,000, 2 mM MgCl_2_, and 10 mM DTT. We harvested the crystals by soaking them briefly in a solution containing 0.1 M MES (pH 6.5), 2 M NaCl, 15% (w/v) PEG 6,000, and 10 mM DTT. The crystals were loop mounted and flash frozen in liquid nitrogen. A complete and highly redundant data set to 2.5 Å resolution was collected at the anomalous peak of Se (*λ* = 0.9794 Å) at the beamline ID14-4 of the European Synchrotron Radiation Facility (ESRF). The diffraction data were processed using XDS (Kabsch, 2010).

Phases were determined *de novo* using the single-wavelength anomalous dispersion (SAD) technique based on the anomalous signal from SeMet incorporated into the protein. Selenium sites were located and experimental maps were calculated using AutoSol in the software suite Phenix (Terwilliger et al., 2009). Models were built using the program COOT (Emsley and Cowtan, 2004), and refinement carried out by Phenix.refine (Adams et al., 2010) using data in the range of 20–2.5 Å. The final model was validated by MolProbity (Chen et al., 2010). All subsequent structure analyses and figure generations were carried out using PyMol (http://www.pymol.org) or CCP4mg (McNicholas et al., 2011). The details of data collection and refinement statistics are summarized in Table 1.

**TABLE 1:** Data collection and refinement statistics.

Wavelength (Å)	0.9794
Resolution range (Å)	20–2.50 (2.59–2.50)
Space group	I 4 2 2
Unit cell: *a*, *b*, *c* (Å); *α*, *β*, *γ* (°)	190.004, 190.004, 175.275; 90, 90, 90
Total reflections	733,895 (39,598)
Unique reflections	49,419 (2,815)
Multiplicity	14.9 (14.1)
Completeness (%)	89.20 (51.46)
Mean I/sigma(I)	20.47 (1.63)
R-merge	0.1335 (1.691)
R-meas	0.1382 (1.754)
R-pim	0.03551 (0.4599)
CC_1/2_	0.999 (0.601)
CC*	1.00 (0.866)
	
Reflections used in refinement	49,410 (2,816)
Reflections used for R-free	1,992 (104)
R-work	0.1893
R-free	0.2154
Number of non-hydrogen atoms in proteins	6,184
Number of non-hydrogen atoms in solvent	90
RMS (bonds)	0.004
RMS (angles)	0.75
Ramachandran favored (%)	98.01
Ramachandran allowed (%)	1.99
Ramachandran outliers (%)	0.00

Statistics for the highest-resolution shell are shown in parentheses.

### Isothermal titration calorimetry (ITC)

All ITC measurements were conducted on a MicroCal PEAQ-ITC microcalorimeter (Malvern Panalytical). Purified SAP102, Sec8c, and Sec8c-Δ958–971 proteins were dialyzed overnight against a buffer composed of 20 mM HEPES (pH 7.5), 100 mM NaCl, and 1 mM DTT. Protein concentration was determined on a UV spectrometer at 280 nm, and the calculation was based on the extinction coefficient of each protein (Sec8c/Sec8c-Δ958–971: 35,870 M^−1^cm^−1^; PDZ1-PDZ2: 5,960 M^−1^cm^−1^; PDZ1: 1,490 M^−1^cm^−1^; PDZ2: 4,470 M^−1^cm^−1^; PDZ3: 2,980 M^−1^cm^−1^). For ITC measurements, the reaction chamber and the injection syringe contained 250 µL of 30 µM Sec8c (or Sec8c-Δ958–971) and 45 µL of 300 µM SAP102, respectively. All titration experiments consisted of one initial 0.4 µL injection followed by 18 consecutive 2 µL injections with a duration of 4 s each and an interval of 120 s between two consecutive injections. The resulting data were analyzed with the MicroCal PEAQ-ITC Analysis Software (Malvern Panalytical, Version 1.22) using the one-set-of-site fitting model. Non-linear least square fitting using one binding site model was used to calculate the association constant (Ka). Dissociation constants (Kd) were calculated according to the formula Kd = 1/Ka. Each measurement was carried at least twice, and the most representative one were shown in the figures. All ITC data were the sample titration (protein to protein) subtracted by the control titration (protein to buffer).

### Size exclusion chromatography (SEC)

For using SEC to detect protein complex formation, purified Sec8c or Sec8c-Δ958–971 (1 mg/mL) was mixed with various SAP102 truncations (molar ratio 1:2). The final sample volume was approximately 500 µL for mixtures with PDZ1-PDZ2 or PDZ2 and 80 µL for those with PDZ1 and PDZ3. After incubation at 4°C for 30 min, the mixtures were loaded onto a Superdex 200 Increase 10/300 GL column and ran using the same sample buffer containing 20 mM HEPES (pH 7.5), 100 mM NaCl, and 1 mM DTT. The peak fractions were loaded onto an SDS-PAGE gel to check the presence of either Sec8 or SAP102 or both (Supplementary Figure S1).

### *In-vitro* pull-down assays

For pull-down experiments between Sec8 and SAP102, 0.5 mL of purified MBP-His_10_-Sec8c or MBP-His_10_-Sec8c-Δ958–971 (1 mg/mL) was mixed with excessive amount of purified SAP102 truncations (molar ratio 1:2–5) in the binding buffer containing 20 mM HEPES (pH 7.5), 100 mM NaCl, 1 mM DTT, and 1% (v/v) glycerol. The mixtures were incubated with 50–100 µL of prewashed amylose beads under constant tumbling at 4°C for 1–2 h. The beads were subsequently washed using 4 × 200 µL of the same binding buffer to remove non-specifically bound SAP102. Bound proteins on the beads were checked on an SDS-PAGE gel. As negative controls to detect non-specific interactions, the same set of pull-downs was also performed in parallel using the MBP-His_10_ tag mixed with various SAP102 truncations.

### Protein structural prediction, modeling and visualization

The structural predictions of the full-length rat SAP102 and exocyst subunits were obtained from the AlphaFold Protein Structure Database (Jumper et al., 2021) (https://alphafold.ebi.ac.uk). All other predicted models were generated using the AlphaFold2 neural-network implemented within the freely accessible ColabFold pipeline (Mirdita et al., 2022). Structure analyses and figure generations were carried out using PyMol (http://www.pymol.org) or CCP4mg (McNicholas et al., 2011).

## Results

### Crystal structure of the C-terminal region of rat Sec8

**FIGURE 1:**
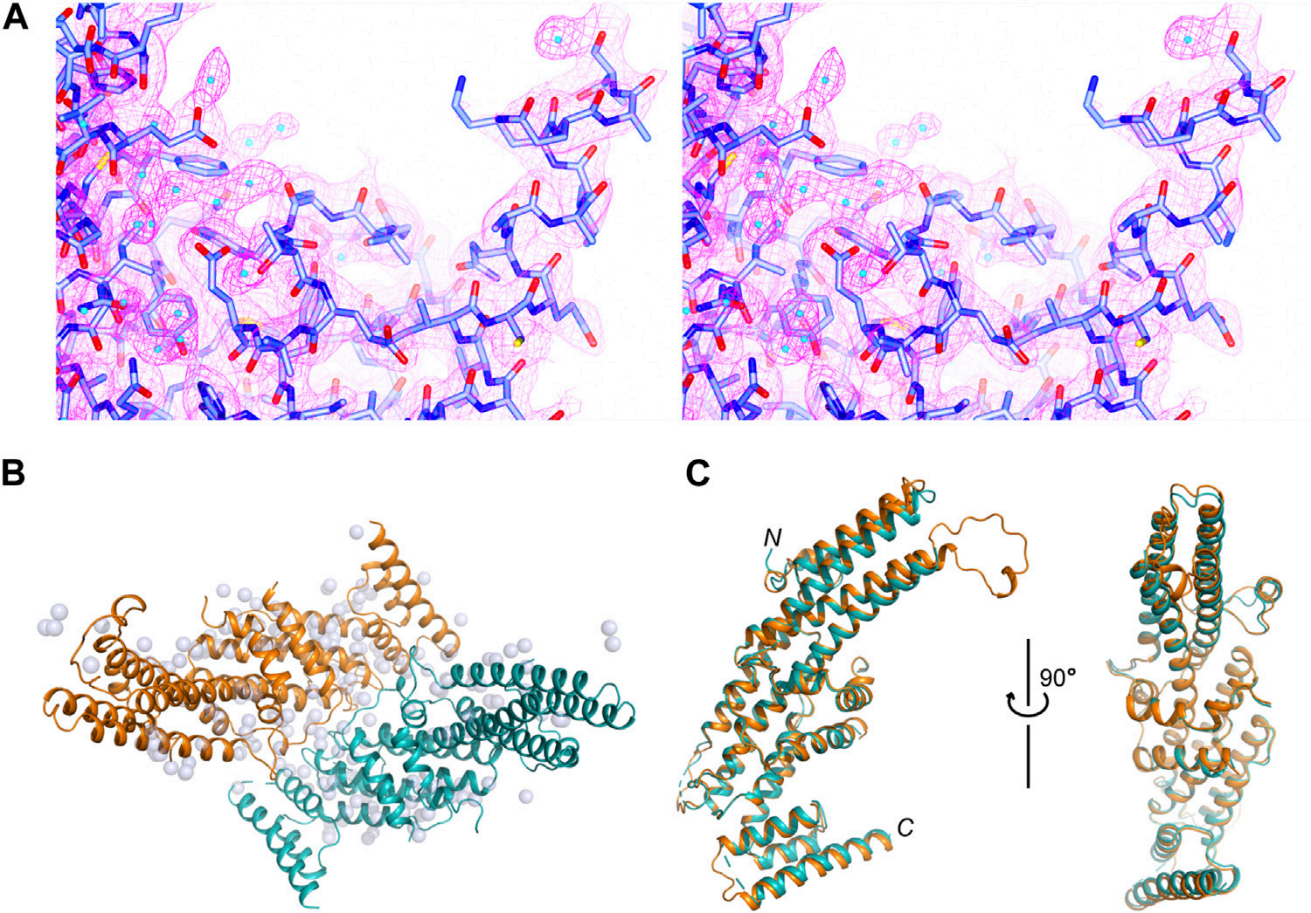
Crystal structure of the C-terminal region of rat Sec8. **(A)** Stereo views of the 2*F*_*o*_ *−F*_*c*_ electron density map contoured at 1.5 σ level around the C-terminus of Sec8. Water molecules are shown as cyan-colored spheres. **(B)** Packing of the two Sec8c molecules in the asymmetric unit. Sec8 molecules are shown as ribbon diagrams in orange and teal, and water molecules as grey spheres. **(C)** Superposition of the two Sec8 molecules from the asymmetric unit shown in **(B)**.

We determined a crystal structure for the C-terminal region of *Rattus norvegicus* Sec8 (aa556–975), which will be called Sec8c hereafter. The structure was refined to 2.5 Å resolution, with excellent 2*F*_*o*_-*F*_*c*_ electron density maps (Figure 1A). The crystal belongs to space group *I* 422 (a = b = 190.004 Å, c = 175.275 Å), with two molecules in the asymmetric unit (Figure 1B). The final structure was refined to *R*_*work*_ and *R*_*free*_ of 18.93% and 21.54%, respectively (Table 1). One molecule contains aa557–661 and 674–967 of Sec8, and the other contains aa556–662, 675–732, 754–802, 808–936 and 944–967. Except for the flexible loops, the two Sec8 molecules in the asymmetric unit are essentially the same, with an average root-mean-square deviation (RMSD) of approximately 0.5 Å for all aligned backbone atoms (Figure 1C). Additionally, there are 90 water molecules confidently built in the refined model.

### Domain organization and structural analyses of SAP102

**FIGURE 2:**
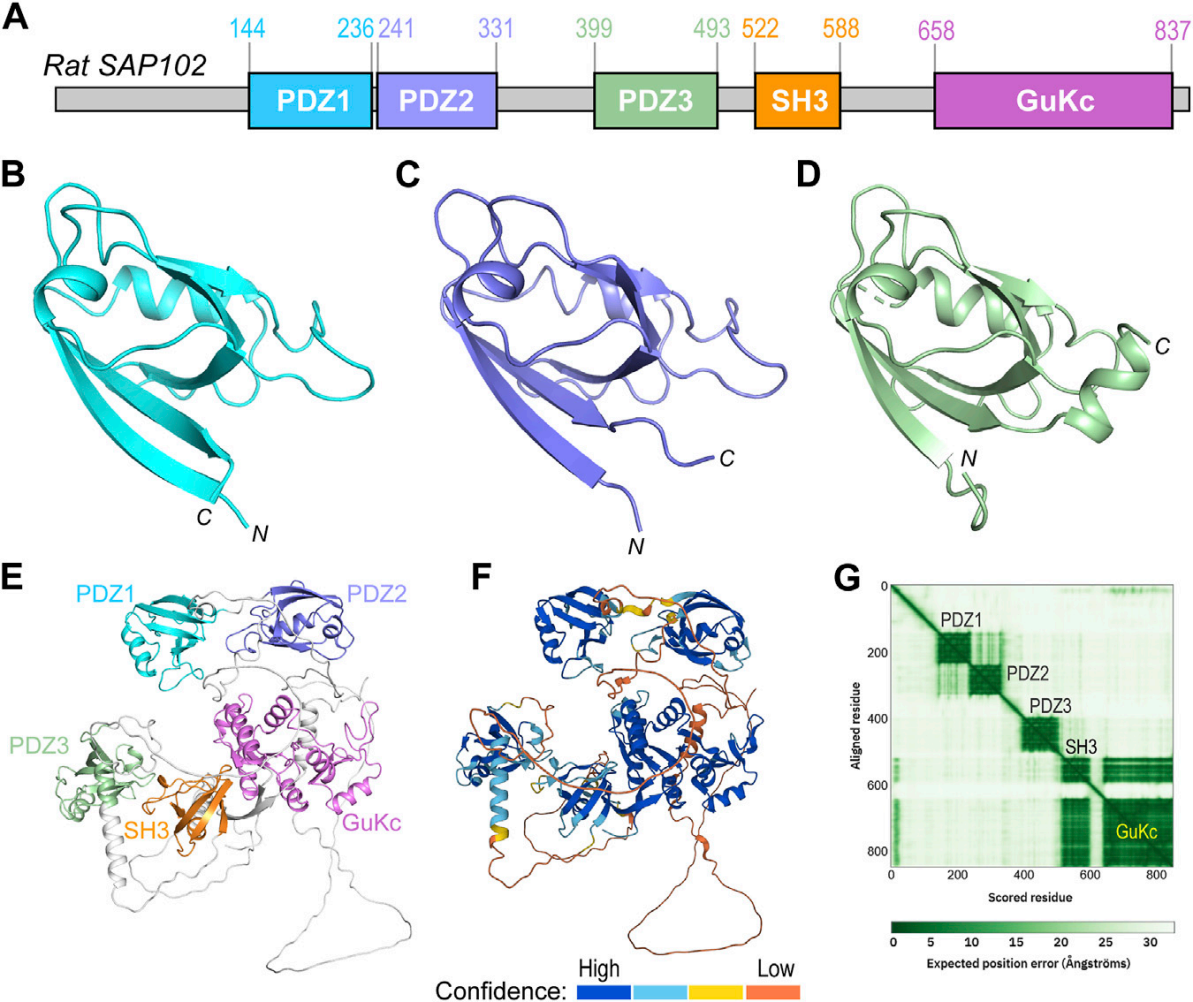
Domain organization and structural analyses of SAP102. **(A)** Domain organization of rat SAP102. Boundaries of each domain are shown above the diagram. **(B–D)** Ribbon diagrams of the crystal structures of PDZ1 [**(B)**, PDB code: 2I1N], PDZ2 [**(C)**, PDB code: 2FE5], and PDZ3 [**(D)**, PDB code: 3JXT]. **(E)** Ribbon diagram of full-length SAP102 model generated by AlphaFold2, with individual domains shown in the same color as those in **(A)**. **(F)** The same model of SAP102 as in **(E)** colored by per-residue confidence scores. **(G)** Predicted aligned errors generated by AlphaFold2 for the SAP102.

*Rattus norvegicus* SAP102 consists of 849 residues, which fold into five domains (Figure 2A). The first three domains are PDZs (PDZ1-3), with PDZ1 and PDZ2 being tightly connected by a short linker (aa237–240). PDZ3 (aa399–493) is far apart and loosely linked to the first two PDZ domains by a long loop. Crystal structures of all these PDZ domains have been reported (Figures 2B–D) (Doyle et al., 1996; Elkins et al., 2007; Camara-Artigas et al., 2010; Sainlos et al., 2011; Bach et al., 2012; Sainlos et al., 2013; Zeng et al., 2016; Camara-Artigas et al., 2019; Rodzli et al., 2020; Fukata et al., 2021). The predicted model of full-length SAP102 from the AlphaFold Protein Structure Database (https://alphafold.ebi.ac.uk) also shows a compact folding of each domain (Figure 2E), which is supported by the per-residue confidence scores in these domains in contrast to the low scores in the loops (Figure 2F). The 2D plot of the predicted aligned errors (PAE) further demonstrates that PDZ1 and PDZ2 are closely linked together by a short linker (Figure 2G).

### Sec8 binds specifically to the PDZ2 domain of SAP102

**FIGURE 3:**
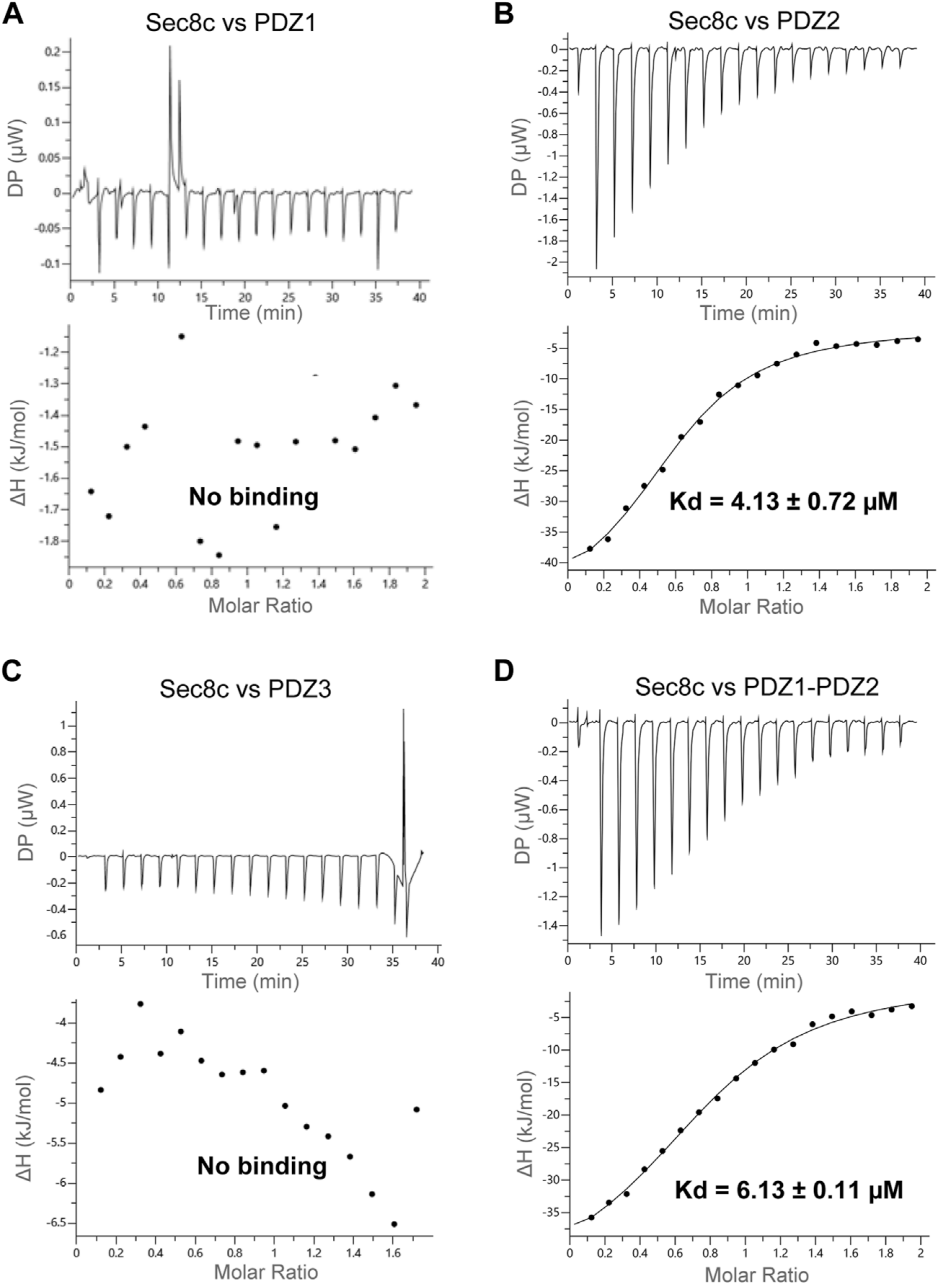
ITC measurements of the interaction between Sec8c and the PDZ domains of SAP102. **(A)** No interaction was detected between Sec8c and PDZ1 of SAP102. **(B)** Sec8c bound PDZ2 of SAP102 robustly, with a dissociation constant (Kd) of approximately 4 µM. **(C)** Sec8c did not bind PDZ3 of SAP102. **(D)** Sec8c bound PDZ1-PDZ2 of SAP102 with a Kd of approximately 6 µM.

Previous studies showed that Sec8 binds each of the three PDZ domains of SAP102, but with variable affinities (Sans et al., 2003). To examine their interaction and quantitate their binding affinity, we generated expression constructs of individual PDZ domains, as well as the tandem PDZ1-PDZ2. We carried out ITC experiments using recombinant proteins purified from bacteria. The results showed no detectable interaction of Sec8c with either PDZ1 or PDZ3 (Figures 3A, C), whereas Sec8c bound PDZ2 roubustly, with a Kd of approximately 4 µM (Figure 3B). The tandem PDZ1-PDZ2 also bound to Sec8c, with a similar affinity to that of PDZ2 (Figure 3D).

**FIGURE 4:**
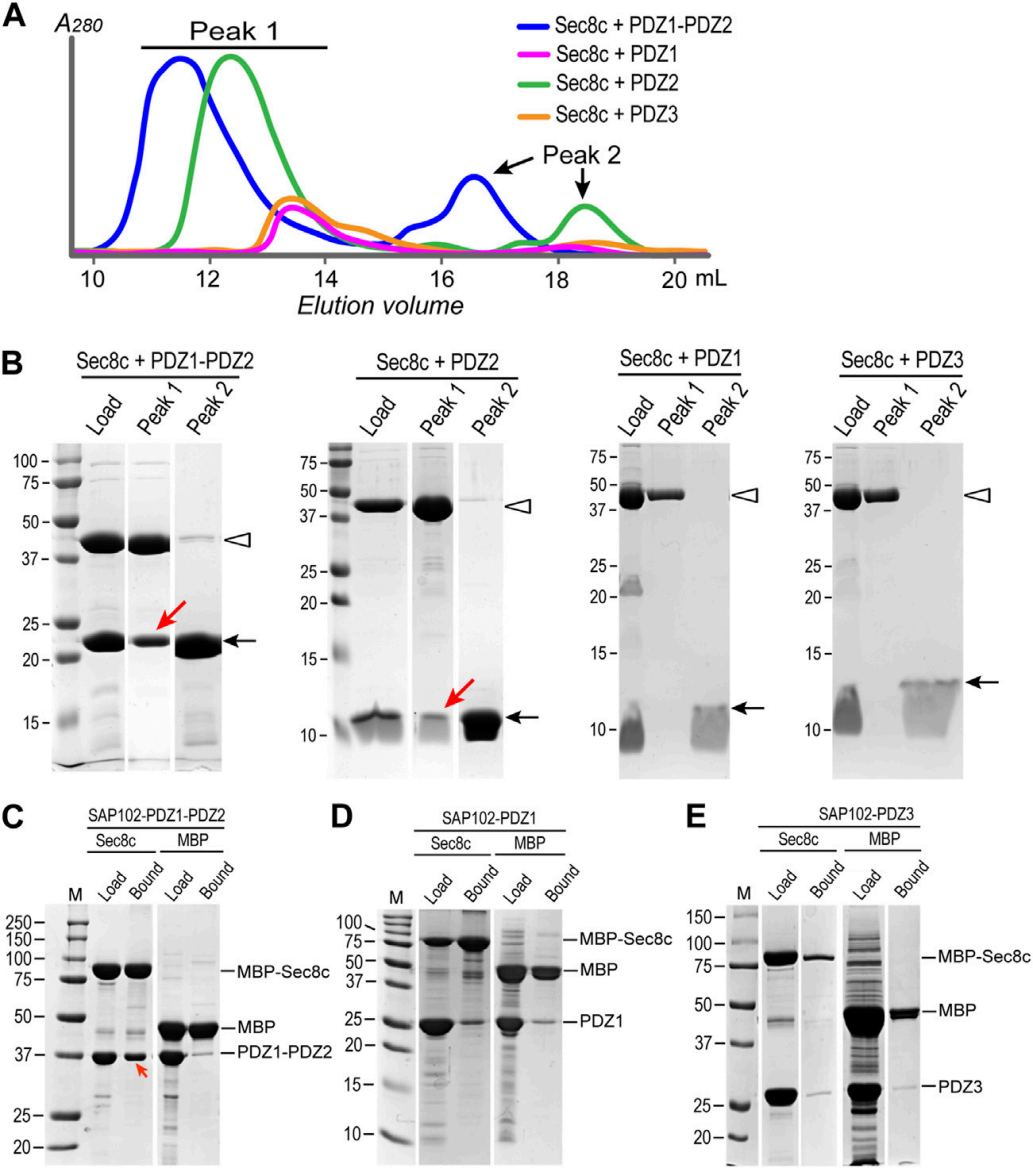
SEC experiments and *in vitro* pull-downs confirm that Sec8c binds PDZ2, but not PDZ1 or PDZ3, of SAP102. **(A)** SEC elution profiles of the mixtures of Sec8c with SAP102 (molar ratio: 1:2) from a Superdex 200 Increase 10/300 GL column. **(B)** Load and samples from peaks 1 and 2 checked on SDS PAGE gels. Positions of Sec8c and SAP102 PDZ domains are marked by hollow arrowheads and arrows, respectively. Red arrows indicate PDZ2 and PDZ1-PDZ2 of SAP102 coeluted with Sec8c in peak 1. The full SDS-PAGE gels for Sec8c-PDZ1-PDZ2 and Sec8c-PDZ2 are shown in Supplementary Figure S1. **(C)** MBP-His_10_-Sec8c pulled down His_6_-SUMO-SAP102-PDZ1-PDZ2 robustly. **(D,E)** Sec8c failed to pull down individual PDZ1 **(D)** or PDZ3 **(E)**. MBP-His_10_ was included as a negative control in each pull-down experiment.

To verify the interaction between Sec8 and SAP102, we further performed SEC experiments for the same set of combinations. In each case, we mixed Sec8c and SAP102 with a molar ratio of 1:2. The mixtures were loaded onto a Superdex 200 Increase 10/300 GL column. Two elution peaks were observed for each mixture, in which the earlier eluted peak (i.e., peak 1) should generally contain a larger protein than the later peak (i.e., peak 2) (Figure 4A). Mixed samples as well as the peak fractions were checked on SDS-PAGE gels (Figure 4B; Supplementary Figure S1). The results revealed that neither PDZ1 nor PDZ3 was coeluted with Sec8c in peak 1. However, both PDZ2 and PDZ1-PDZ2 were coeluted with Sec8c in the first peak, and excessive non-bound PDZ domains were also eluted in peak 2 as they have a much lower molecular weight than that of the complex. These data further confirm that Sec8 preferentially bound to PDZ2.

To further check whether PDZ1 and PDZ3 of SAP102 indeed do not interact with Sec8, we additionally carried out *in vitro* pull-down assays. Purified MBP-His_10_-Sec8c was mixed with excessive His_10_-Sumo-SAP102, and the mixture was incubated with amylose beads. After extensive washes, bound proteins on the beads were checked on SDS-PAGE gels. The results demonstrated that the tandem PDZ1-PDZ2 of SAP102 were robustly pulled down by MBP-His_10_-Sec8c (Figure 4C). However, neither PDZ1 nor PDZ3 alone could be pulled down by Sec8c, as the amounts bound to the column were roughly the same as the negative control in which only the MBP-His_10_ tag were used to mix with SAP102 (Figures 4D, E).

### Sec8 is the only exocyst subunit containing a long helical spacer

**FIGURE 5:**
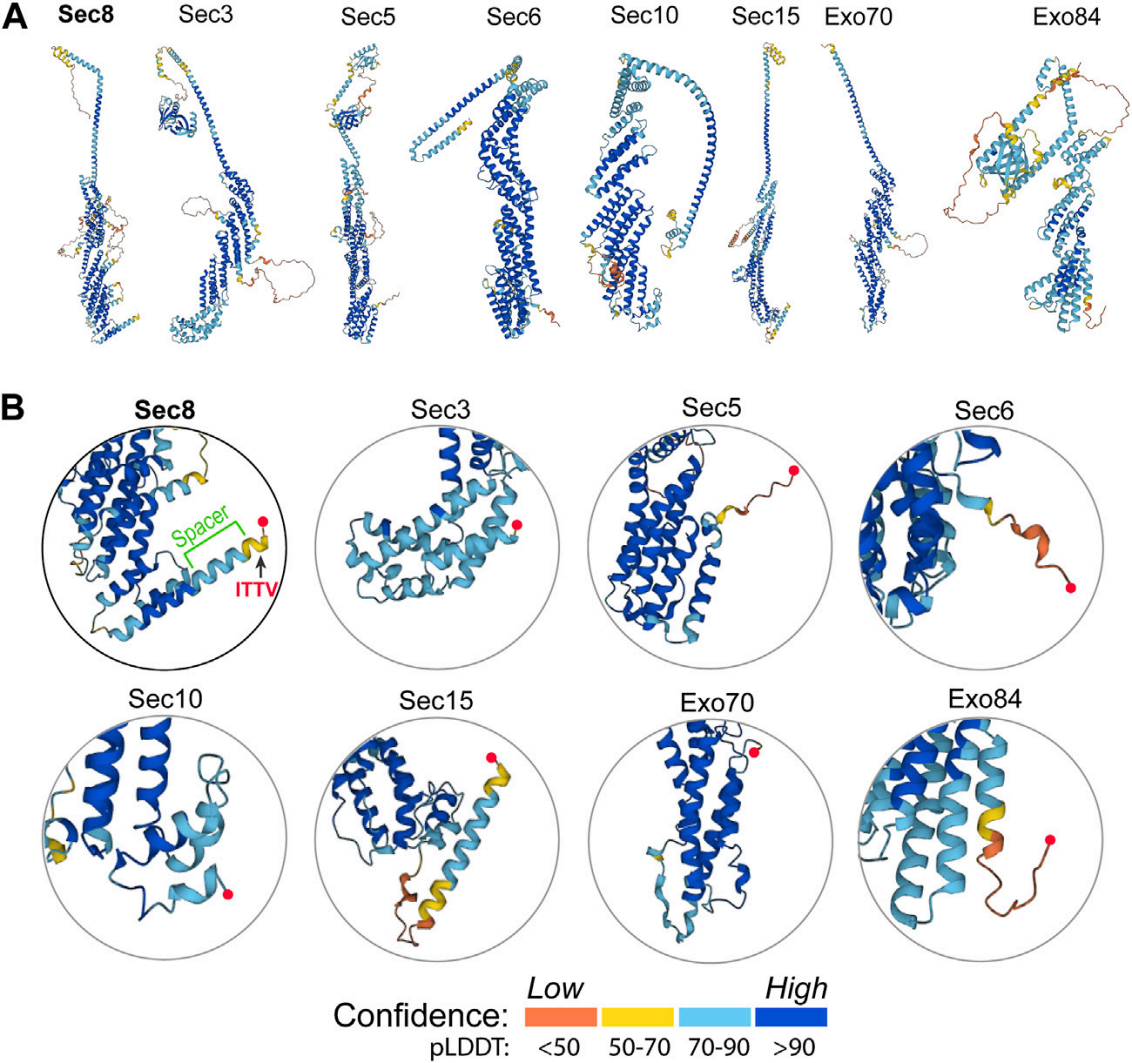
Structural analyses of the AlphaFold models of all eight exocyst components. **(A)** Ribbon diagrams of the predicted models of all exocyst subunits taken from the AlphaFold Protein Structure Database (https://alphafold.ebi.ac.uk). The structures were colored by model confidence. **(B)** Enlarged views of the C-terminal region of the exocyst subunits. The C-terminal end in each model is marked by a red sphere. Sec8 contains a long C-terminal helix, half of which protrudes out from the core to connect to the PDZ-binding ITTV motif as a “spacer”.

To better understand how Sec8 interacts with SAP102, we further checked and compared the folding and structure of Sec8 with other exocyst subunits. Predicted models of all the eight exocyst components were taken from the AlphaFold Protein Structure Database (https://alphafold.ebi.ac.uk). Ribbon diagrams of these models were generated and shown side-by-side for comparison (Figure 5A). The enlarged views show significant differences in their C-terminal regions (Figure 5B). In three exocyst subunits (Sec3, Sec10 and Exo70) the C-terminal ends are packed tightly against the helical core of their structures. The other three subunits (Sec5, Sec6 and Exo84) contain a long flexible loop at their C-terminus. Sec15 is the only one that also has a long helix at its C-terminal end, but its C-terminal tip is very close to the folded helical bundle of the protein. Overall, Sec8 is the only exocyst subunit that contains a rigid long spacer that bridges the PDZ-binding ITTV motif to the helical core structure.

### The spacer in Sec8 is essential for its interaction with SAP102

**FIGURE 6:**
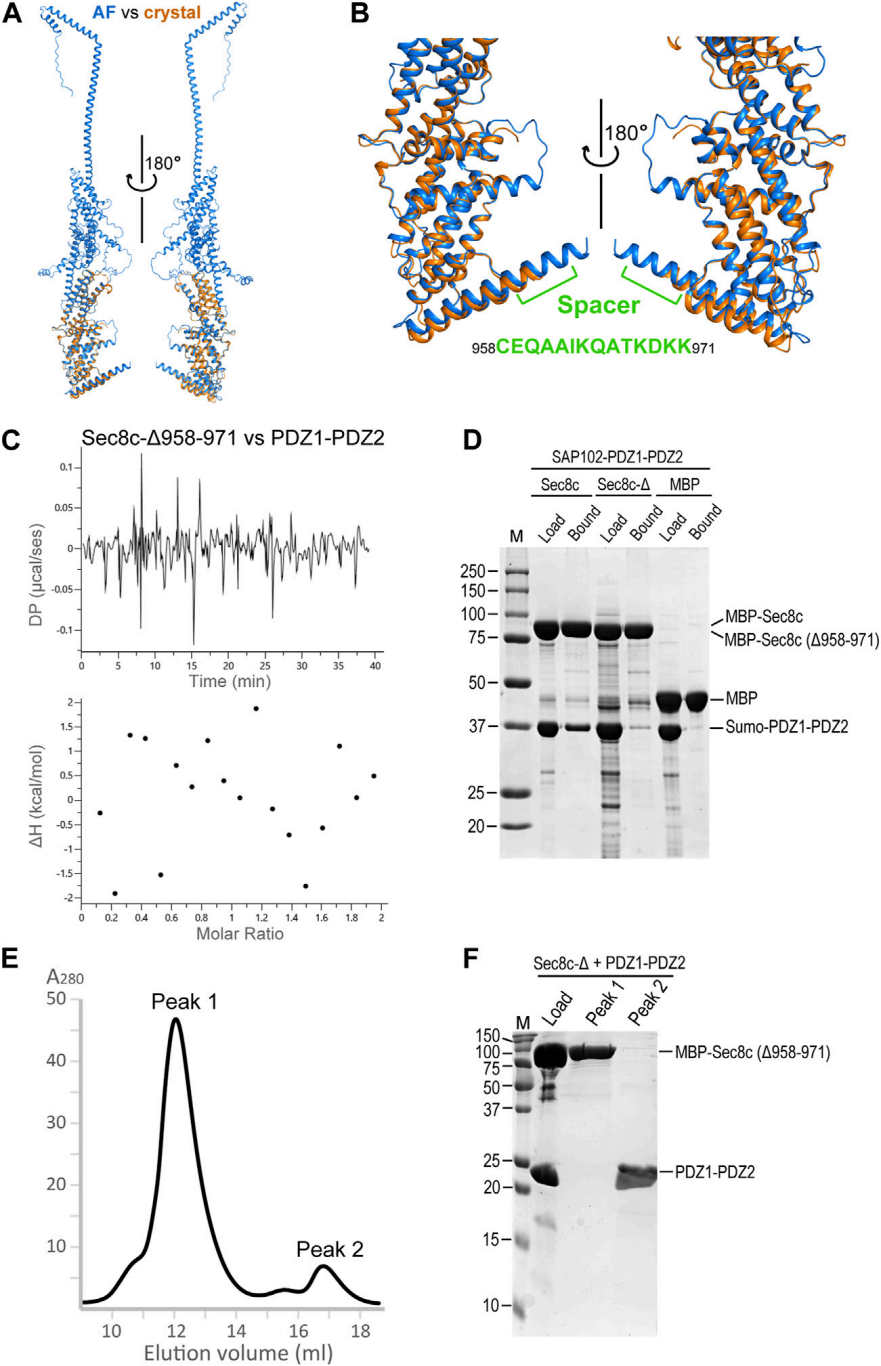
The C-terminal spacer of Sec8 is essential for its interaction with SAP102-PDZ2. **(A)** Superposition of the Sec8c crystal structure (orange) on top of the AlphaFold model of full-length Sec8 (blue). **(B)** Enlarged views of the superposition in **(A)**. The spacer is indicated and the corresponding sequence is shown. **(C)** ITC data shows that Sec8c-Δ958–971 did not bind PDZ1-PDZ2. **(D)** Only Sec8c, but not Sec8c-Δ958–971, could pull down SAP102-PDZ1-PDZ2. **(E)** SEC elution profile of the mixture of MBP-His_10_-Sec8c (Δ958–971) with SAP102-PDZ1-PDZ2 (molar ratio: 1:2) on a Superdex 200 Increase 10/300 GL column. **(F)** Samples for the loaded mixture and fractions from peaks 1 and 2 on an SDS PAGE gel.

Comparison of the crystal structure of Sec8c with the AlphaFold model of full-length Sec8 shows that, except for some of the flexible loops, the two models are essentially the same and can be excellently superimposed on top of each other (Figure 6A). The spacer is slightly shorter in the crystal structure, which was due to the lack of visible electron densities from residue K968 to the C-terminus. In the AlphaFold model the helix is predicted to extend all the way to the last residue at the C-terminus (Figure 6B). We checked whether the spacer in Sec8 is necessary for its binding to SAP102 using three independent methods. Our ITC data showed no detectable interaction at all between Sec8c-Δ958–971 and SAP102-PDZ1-PDZ2 (Figure 6C). Our *in vitro* pull-down results also showed that Sec8c lacking the spacer (Δ958–971) failed to pull down PDZ1-PDZ2 (Figure 6D). Consistently, SEC results of the mixture further demonstrated that SAP102-PDZ1-PDZ2 was eluted separately in a later peak, and no stable complex was formed between it and Sec8c-Δ958–971 (Figures 6E, F).

## Discussion

*N*-methyl-D-aspartate receptors (NMDARs) are sorted from the ER and the Golgi apparatus to the synapse with the help of the exocyst complex and SAP102 (Sans et al., 2003). SAP102 binds specifically to one of the exocyst subunits, Sec8, via its PDZ domains. Here we report the crystal structure of the C-terminal half of *R*. *norvegicus* Sec8 (Figure 1). SAP102, like PSD-95, consists of five domains, with PDZ1 and PDZ2 forming a tandem structure tightly linked by a short connecting linker (Figure 2). Our ITC data revealed that Sec8c binds specifically to PDZ2 of SAP102, but shows no detectable interaction with either PDZ1 or PDZ3 (Figure 3). The selective interaction of Sec8 with SAP102-PDZ2 was further confirmed by SEC results (Figures 4A, B). Additional *in vitro* pull-down assays also did not detect any significant interaction of PDZ1 and PDZ3 with Sec8c (Figures 4C–E). All these together demonstrate that Sec8 binds specifically to SAP102-PDZ2. This result is a bit different from what was reported previously, where a pull-down assay demonstrated that despite Sec8 interacted with PDZ2 most robustly, it also bound to PDZ1 and PDZ3 to a lesser degree (Sans et al., 2003). The discrepancy could be due to the different experiment setups (*in vitro* vs. *in vivo*), variations in construct length (truncated CTD vs. full-length of Sec8), or the difference in the fusion tags used. Nevertheless, both studies suggest SAP102-PDZ2 bears the main binding site of Sec8.

Comparison of 3D models of all exocyst subunits shows that Sec8 contains a unique long helix at its C-terminal end. The C-terminal half of this helix protrudes out from the compact structural core of Sec8. It forms a rigid spacer bridging the PDZ-binding ITTV motif with the helical bundles in the structure. The spacer is present only in Sec8 but not in any other exocyst subunits (Figure 5). Deletion of the spacer completely abolished the interaction of Sec8 with SAP102, as demonstrated by our ITC, pull-down and SEC data (Figure 6). We did not observe any abnormal phenomenon during the purification of the spacer-deleting construct, which suggests that such deletion does not cause collapse of the overall folding of Sec8c. However, we could not rule out the possibility that such deletion might make subtle changes to the local structure around the deletion site or prevent the ITTV motif from adopting a favorable orientation for PDZ binding.

**FIGURE 7:**
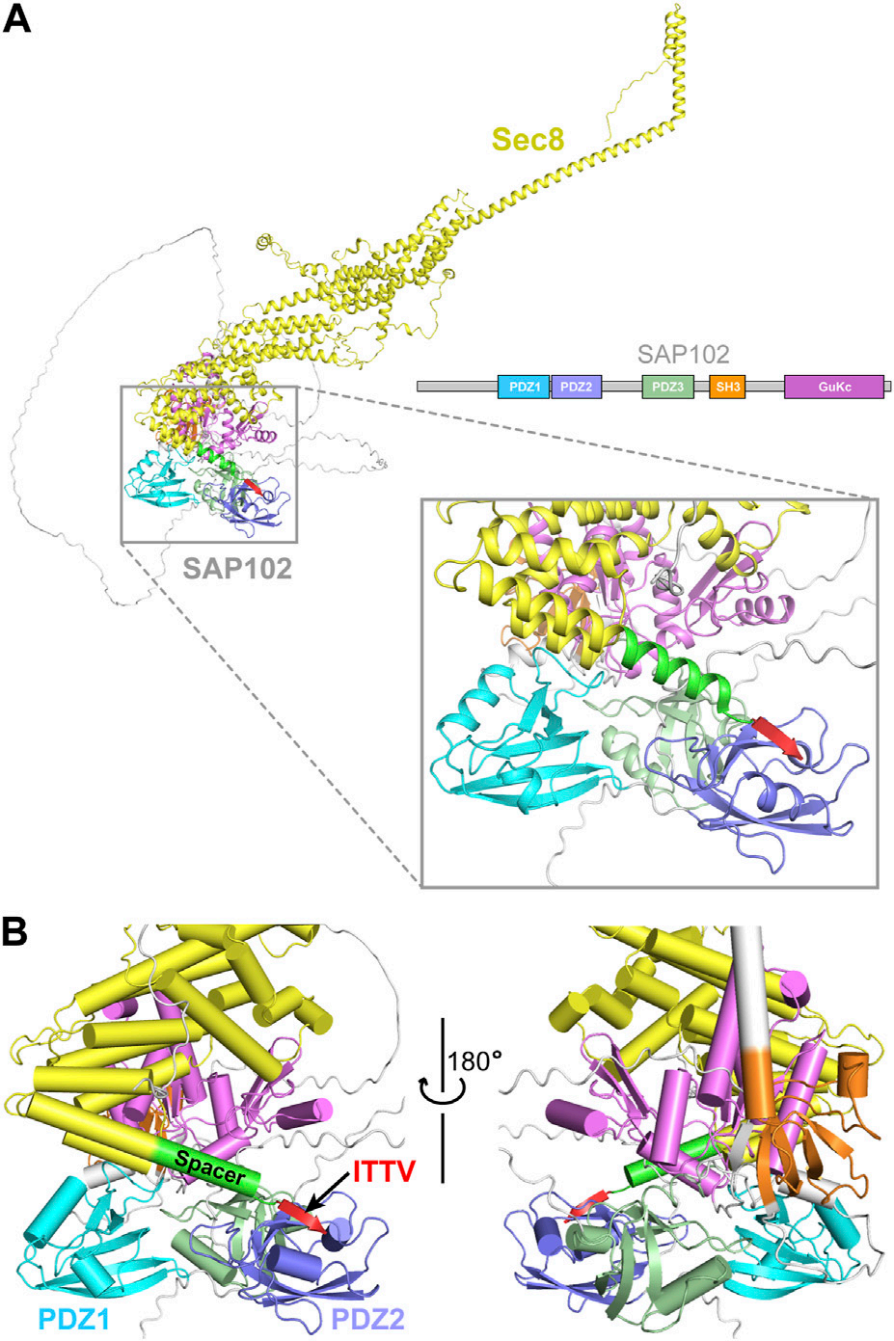
Prediction of the interaction between Sec8 and SAP102. **(A)** AlphaFold model of full-length Sec8 (yellow) in complex with full-length SAP102 with its individual domains differently colored. **(B)** Enlarged views of the interaction interface between the C-terminus of Sec8 and SAP10-PDZ1-PDZ2. The four residues “ITTV” (red) at the C-terminal tip of Sec8 attach to the edge of the β sheet in PDZ2 and become an integral part of the sheet. The 14-residue spacer (green) delivers the ITTV motif to its targeting site on PDZ2, preventing the clash of the helically bundled core (yellow) of Sec8 with SAP102.

3D modeling by the AlphaFold2 ColabFold pipeline using Sec8 and SAP102 generated a structural model for their complex (Mirdita et al., 2022) (Figure 7A). In the model, the C-terminal ITTV motif forms a β strand, which docks to the edge of the β sheet in PDZ2 and becomes an integral part of the sheet (Figure 7B). The 14-residue helical spacer (aa958–971) seems to provide a buffering zone that prevents the clash of the helically bundled core of Sec8 with the multi-domain SAP102. This would explain why deletion of the spacer abolished their interaction.

Why does Sec8 preferentially binds to the PDZ2 domain of SAP102? Notably, crystal structures of the three PDZ domains show that they adopt a similar conformation, with overall RMSDs of approximately 0.6 Å for all backbone atoms in the aligned core structures (Figures 2B–D). However, despite the similarity in the overall folding of the three domains, there are still substantial differences in their primary sequences, with only approximately 45% identity among them. Some subtle differences in the ITTV binding site may confer the binding specificity of Sec8 to PDZ2, but not to PDZ1 or PDZ3 of SAP102. This is supported by the AlphaFold2 predicted model in which the ITTV motif in Sec8 selectively binds to PDZ2 in the full-length SAP102 (Figure 7A). The rigid spacer sandwiched between the ITTV motif and the helical rod of Sec8 might serve to control the relative orientation of the two proteins. This may allow the coordination of the whole exocyst complex with SAP102 so as to facilitate effective sorting and trafficking of NMDA receptors from the ER and the Golgi apparatus to neuronal synapses.

## Data Availability

Coordinate and structure factor of Sec8c generated in this study can be found in the Protein Data Bank (https://www.rcsb.org/structure/8AY2) under the accession code 8AY2.

## References

[B1] Adams P. D. Afonine P. V. Bunkoczi G. Chen V. B. Davis I. W. Echols N. (2010). Phenix: A comprehensive python-based system for macromolecular structure solution. Acta Crystallogr. D. Biol. Crystallogr. 66, 213–221. 10.1107/S0907444909052925 20124702 PMC2815670

[B2] Bach A. Clausen B. H. Moller M. Vestergaard B. Chi C. N. Round A. (2012). A high-affinity, dimeric inhibitor of PSD-95 bivalently interacts with PDZ1-2 and protects against ischemic brain damage. Proc. Natl. Acad. Sci. U. S. A. 109, 3317–3322. 10.1073/pnas.1113761109 22343531 PMC3295328

[B3] Camara-Artigas A. Murciano-Calles J. Gavira J. A. Cobos E. S. Martinez J. C. (2010). Novel conformational aspects of the third PDZ domain of the neuronal post-synaptic density-95 protein revealed from two 1.4A X-ray structures. J. Struct. Biol. 170, 565–569. 10.1016/j.jsb.2010.03.005 20227506

[B4] Camara-Artigas A. Murciano-Calles J. Martinez J. C. (2019). Conformational changes in the third PDZ domain of the neuronal postsynaptic density protein 95. Acta Crystallogr. D. Struct. Biol. 75, 381–391. 10.1107/S2059798319001980 30988255

[B5] Chen V. B. Arendall W. B. 3rd Headd J. J. Keedy D. A. Immormino R. M. Kapral G. J. (2010). MolProbity: all-atom structure validation for macromolecular crystallography. Acta Crystallogr. D. Biol. Crystallogr. 66, 12–21. 10.1107/S0907444909042073 20057044 PMC2803126

[B6] Doublie S. (1997). [29] Preparation of selenomethionyl proteins for phase determination. Methods Enzymol. 276, 523–530. 10.1016/S0076-6879(97)76075-0 27799112

[B7] Doyle D. A. Lee A. Lewis J. Kim E. Sheng M. MacKinnon R. (1996). Crystal structures of a complexed and peptide-free membrane protein-binding domain: molecular basis of peptide recognition by PDZ. Cell 85, 1067–1076. 10.1016/s0092-8674(00)81307-0 8674113

[B8] Elkins J. M. Papagrigoriou E. Berridge G. Yang X. Phillips C. Gileadi C. (2007). Structure of PICK1 and other PDZ domains obtained with the help of self-binding C-terminal extensions. Protein Sci. 16, 683–694. 10.1110/ps.062657507 17384233 PMC2203335

[B9] Emsley P. Cowtan K. (2004). Coot: model-building tools for molecular graphics. Acta Crystallogr. D. Biol. Crystallogr. 60, 2126–2132. 10.1107/S0907444904019158 15572765

[B10] Fukata Y. Chen X. Chiken S. Hirano Y. Yamagata A. Inahashi H. (2021). LGI1-ADAM22-MAGUK configures transsynaptic nanoalignment for synaptic transmission and epilepsy prevention. Proc. Natl. Acad. Sci. U. S. A. 118, e2022580118. 10.1073/pnas.2022580118 33397806 PMC7826393

[B11] Jumper J. Evans R. Pritzel A. Green T. Figurnov M. Ronneberger O. (2021). Highly accurate protein structure prediction with AlphaFold. Nature 596, 583–589. 10.1038/s41586-021-03819-2 34265844 PMC8371605

[B12] Kabsch W. (2010). Xds. Acta Crystallogr. D. Biol. Crystallogr. 66, 125–132. 10.1107/S0907444909047337 20124692 PMC2815665

[B13] McNicholas S. Potterton E. Wilson K. S. Noble M. E. (2011). Presenting your structures: the CCP4mg molecular-graphics software. Acta Crystallogr. D. Biol. Crystallogr. 67, 386–394. 10.1107/S0907444911007281 21460457 PMC3069754

[B14] Mirdita M. Schutze K. Moriwaki Y. Heo L. Ovchinnikov S. Steinegger M. (2022). ColabFold: making protein folding accessible to all. Nat. Methods 19, 679–682. 10.1038/s41592-022-01488-1 35637307 PMC9184281

[B15] Riefler G. M. Balasingam G. Lucas K. G. Wang S. Hsu S. C. Firestein B. L. (2003). Exocyst complex subunit sec8 binds to postsynaptic density protein-95 (PSD-95): A novel interaction regulated by cypin (cytosolic PSD-95 interactor). Biochem. J. 373, 49–55. 10.1042/BJ20021838 12675619 PMC1223477

[B16] Rodzli N. A. Lockhart-Cairns M. P. Levy C. W. Chipperfield J. Bird L. Baldock C. (2020). The dual PDZ domain from postsynaptic density protein 95 forms a scaffold with peptide ligand. Biophys. J. 119, 667–689. 10.1016/j.bpj.2020.06.018 32652058 PMC7399497

[B17] Sainlos M. Iskenderian-Epps W. S. Olivier N. B. Choquet D. Imperiali B. (2013). Caged mono- and divalent ligands for light-assisted disruption of PDZ domain-mediated interactions. J. Am. Chem. Soc. 135, 4580–4583. 10.1021/ja309870q 23480637

[B18] Sainlos M. Tigaret C. Poujol C. Olivier N. B. Bard L. Breillat C. (2011). Biomimetic divalent ligands for the acute disruption of synaptic AMPAR stabilization. Nat. Chem. Biol. 7, 81–91. 10.1038/nchembio.498 21186349

[B19] Sans N. Prybylowski K. Petralia R. S. Chang K. Wang Y. X. Racca C. (2003). NMDA receptor trafficking through an interaction between PDZ proteins and the exocyst complex. Nat. Cell Biol. 5, 520–530. 10.1038/ncb990 12738960

[B20] TerBush D. R. Maurice T. Roth D. Novick P. (1996). The Exocyst is a multiprotein complex required for exocytosis in *Saccharomyces cerevisiae* . EMBO J. 15, 6483–6494. 10.1002/j.1460-2075.1996.tb01039.x 8978675 PMC452473

[B21] TerBush D. R. Novick P. (1995). Sec6, Sec8, and Sec15 are components of a multisubunit complex which localizes to small bud tips in *Saccharomyces cerevisiae* . J. Cell Biol. 130, 299–312. 10.1083/jcb.130.2.299 7615633 PMC2199927

[B22] Terwilliger T. C. Adams P. D. Read R. J. McCoy A. J. Moriarty N. W. Grosse-Kunstleve R. W. (2009). Decision-making in structure solution using bayesian estimates of map quality: the PHENIX AutoSol wizard. Acta Crystallogr. D. Biol. Crystallogr. 65, 582–601. 10.1107/S0907444909012098 19465773 PMC2685735

[B23] Zeng M. Shang Y. Araki Y. Guo T. Huganir R. L. Zhang M. (2016). Phase transition in postsynaptic densities underlies formation of synaptic complexes and synaptic plasticity. Cell 166, 1163–1175. 10.1016/j.cell.2016.07.008 27565345 PMC5564291

