# Supplementary material for: Sec8 specifically interacts with the PDZ2 domain of synapse associated protein 102 (SAP102)
Source: Front Cell Dev Biol. 2023 Oct 2;11:1254611. doi: 10.3389/fcell.2023.1254611 (PMC10577314; doi:10.3389/fcell.2023.1254611)

Figure S1. SEC elution profiles and corresponding full SDS-PAGE gels. (A) Sec8c-PDZ1-PDZ. **(B)** Sec8c-PDZ. Arrows indicate the lanes taken out and shown in Figure 4B as well as the elution positions in peak 1 and peak 2 from the SEC column. **(C, D)** The SEC elution profile and the corresponding SDS-PAGE gel for Sec8c-PDZ1 (**C**) and Sec8c-PDZ3 (**D**). To clearly visualize the low amount of proteins, each peak in (C) and (D) was pooled and concentrated to a smaller volume before being loaded onto the SDS gel.

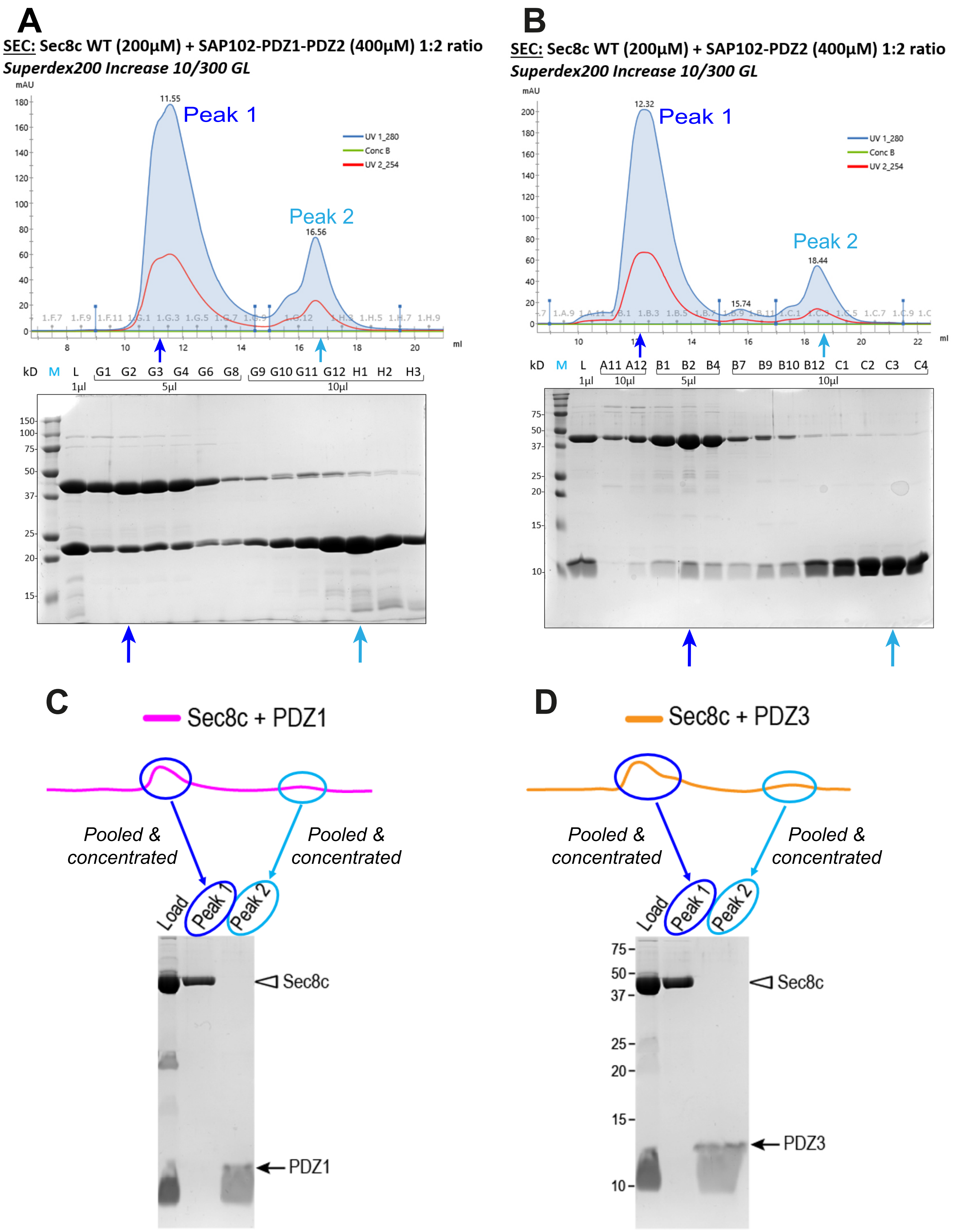

Supplement: Supplementary file 1 [file DataSheet1.PDF]
